# Trends in Complementary and Alternative Medicine Use in Myocardial Infarction

**DOI:** 10.1093/geroni/igaf122.2897

**Published:** 2025-12-31

**Authors:** Audrey Shawley, Najah Khan, Kevin Kozakowski

**Affiliations:** Scripps Health, San Diego, California, United States; Scripps Green Hospital, San Diego, California, United States; Scripps Health, San Diego, California, United States

## Abstract

Complementary and alternative medicine (CAM) is a group of diverse medical and health care systems, practices, and products that are not generally considered part of conventional medicine. There is increasing interest in CAM use among patients with conditions such as myocardial infarction (MI). This study aims to evaluate CAM use among adults with a history of MI. A cross-sectional analysis using data from the Integrated Public Use Microdata Series - National Health Interview Survey from 2002 was performed to assess CAM use in adults with a history of MI. This was the last year CAM data was known to have been collected. Descriptive statistics and Fisher’s exact test were used to analyze the association between any single CAM modality and demographic variables. Of 1,845 respondents with a history of MI, 14 reported using at least one CAM modality. The mean age was 67 years, with other demographic variations. The most commonly used modalities were relaxation and diet therapy. No other associations were identified between demographic variables and CAM use. Though the sample size was limited, this study highlights potential trends and the increased use of CAM in adults with a history of MI. Healthcare providers should be aware of CAM use in patients with MI and discuss its benefits and risks with their patients to ensure safe and well-coordinated care. More rigorous research is needed to determine the effects and long-term benefits of CAM use on cardiovascular morbidity and mortality in patients with a history of MI.

